# Particles from the *Echinococcus granulosus* laminated layer inhibit IL-4 and growth factor-driven Akt phosphorylation and proliferative responses in macrophages

**DOI:** 10.1038/srep39204

**Published:** 2016-12-14

**Authors:** Paula I. Seoane, Dominik Rückerl, Cecilia Casaravilla, Anabella A. Barrios, Álvaro Pittini, Andrew S. MacDonald, Judith E. Allen, Alvaro Díaz

**Affiliations:** 1 Cátedra de Inmunología, Departamento de Biociencias (Facultad de Química) e Instituto de Química Biológica (Facultad de Ciencias), Universidad de la República, Montevideo, Uruguay; 2 Faculty of Biology, Medicine and Health, School of Biological Sciences, University of Manchester, Manchester, UK; 3 Manchester Collaborative Centre for Inflammation Research (MCCIR), University of Manchester, Manchester, UK

## Abstract

Proliferation of macrophages is a hallmark of inflammation in many type 2 settings including helminth infections. The cellular expansion is driven by the type 2 cytokine interleukin-4 (IL-4), as well as by M-CSF, which also controls homeostatic levels of tissue resident macrophages. Cystic echinococcosis, caused by the tissue-dwelling larval stage of the cestode *Echinococcus granulosus*, is characterised by normally subdued local inflammation. Infiltrating host cells make contact only with the acellular protective coat of the parasite, called laminated layer, particles of which can be ingested by phagocytic cells. Here we report that a particulate preparation from this layer (pLL) strongly inhibits the proliferation of macrophages in response to IL-4 or M-CSF. In addition, pLL also inhibits IL-4-driven up-regulation of Relm-α, without similarly affecting Chitinase-like 3 (Chil3/Ym1). IL-4-driven cell proliferation and up-regulation of Relm-α are both known to depend on the phosphatidylinositol (PI3K)/Akt pathway, which is dispensable for induction of Chil3/Ym1. Exposure to pLL *in vitro* inhibited Akt activation in response to proliferative stimuli, providing a potential mechanism for its activities. Our results suggest that the *E. granulosus* laminated layer exerts some of its anti-inflammatory properties through inhibition of PI3K/Akt activation and consequent limitation of macrophage proliferation.

Interleukin (IL)-4 receptor alpha signalling is central to type 2 immune responses. In particular, it drives the activation of macrophages and other myeloid cells in response to IL-4 and IL-13 that is observed at sites of helminth infection^1^. These IL-4Rα-activated macrophages (M(IL-4)) are characterised by up-regulation of Arginase, resistin-like molecule alpha (Relm-α) and Chitinase like 3 (Chil3/Ym1)^1^,^2^,^3^. Over the last five years it has been recognised that in addition to inducing the M(IL-4) phenotype, IL-4 also directly stimulates proliferation of macrophages^4^,^5^. Accordingly, M(IL-4) activation as well as macrophage proliferation, are observed in a range of different helminth infections^1^. In some infections (e.g. *Litomosoides sigmodontis*), proliferative expansion of tissue- resident cells is the dominant mechanism of macrophage accumulation at the site of infection^1^,^5^. In others (e.g. *Schistosoma mansoni*) monocyte recruitment makes the dominant contribution to macrophage numbers but proliferation of macrophages still occurs^1^,^6^,^7^. Proliferation is also essential in homeostatic maintenance of the numbers of resident macrophages in many tissues, typically driven by the growth factor M-CSF^8^,^9^. Indeed, IL-4 and M-CSF seem to act in synergy during helminth infections allowing macrophages to expand beyond their homeostatic levels^5^. Beyond helminth infections, macrophage proliferation driven both by IL-4 and M-CSF is a central factor in a range of chronic inflammatory pathologies^10^,^11^,^12^.

*Echinococcus granulosus* is a platyhelminth cestode belonging to the genus *taenidae.* The larval stage of this species causes cystic echinococcosis in ungulates, in particular sheep, and accidentally in humans^13^,^14^. These larvae, called hydatid cysts, or more correctly hydatids, are bladder-like structures that grow within internal organs (most commonly liver or lungs), reaching up to tens of cm in diameter. The level of inflammation surrounding *E. granulosus* hydatids is usually mild^15^,^16^. In fact, in many cases, the hydatid grows surrounded by a host-derived collagen capsule that is non-infiltrated, or presents cells only distally with respect to the parasite^17^,^18^,^19^. Macrophages in particular appear not to accumulate in the vicinity of the lesion, as determined in human liver infections^20^. This subdued pattern of response is reproduced after experimental infection of mice^21^,^22^. This inflammatory control can fail to varying degrees indicating that it is actively exerted by the parasite. Both in cattle, which is an unsuitable host species^16^,^23^,^24^, as well as in sheep once the parasites die^18^,^19^ macrophages are abundant in the inflammatory infiltrates^16^,^23^,^24^. In experimental mouse infection, macrophages are also prominent in the early response, i.e. before inflammatory control sets in^21^,^22^.

A major feature of larval *Echinococcus* infections is that the parasites shield themselves behind a thick acellular barrier formed mainly by mucins, called the laminated layer (LL)^15^,^25^,^26^. In experimental larval *E. granulosus* infections, the inflammatory response to the establishing parasite resolves once the LL is deployed^21^,^27^. Macrophages that are part of the early infiltrate can be observed to interact directly with the LL surface and to phagocytose LL particles^22^. M(IL-4) activation and macrophage proliferation in *E. granulosus* infection have not yet been analysed. However, they are relevant issues considering the importance of IL-4 in the immune response of natural and experimental hosts in this infection^28^,^29^,^30^. Thus, we aimed to elucidate the relationships between exposure to LL particles, macrophage proliferation, and M(IL-4) activation. For this purpose we used a model of LL-derived particles (pLL). We previously showed that pLL causes unconventional activation of dendritic cells (DCs)^31^. In the present article, we show that pLL inhibits macrophage proliferation in response to IL-4 and M-CSF *in vivo* and *in vitro*. In addition, we report that pLL inhibits the induction of the M(IL-4) marker Relm-α without similar effect on Chil3/Ym1. Mechanistically impairment of both proliferation and expression of Relm-α is likely mediated through inhibition of the phosphatidylinositol 3-phosphate kinase (PI3K) signaling pathway, observed in terms of Akt phosphorylation. Taken together our data suggest that the *E. granulosus* LL and/or materials derived from it actively suppress myeloid cell accumulation through inhibition of PI3K/Akt signalling, supporting the establishment of patent infection.

## Results

### pLL inhibits local proliferation of resident macrophages *in vivo*

Injection of IL-4 complex (IL-4c; recombinant IL-4 bound by an anti-IL-4 antibody for extended bioavailability) in mice is an established model for IL-4-driven macrophage proliferation^4^,^5^,^32^. Also, M-CSF-driven macrophage proliferation can be studied *in vivo* using injection of an M-CSF-Fc fusion protein^5^.

In order to analyse the effect of LL materials on macrophage proliferation, mice were injected i.p. with IL-4c or M-CSF-Fc fusion protein immediately followed by pLL (10 or 30 μg total dry mass) administered by the same route. Twenty-four hours after the injections, peritoneal exudate cells (PEC) were analysed by flow cytometry. In addition, pleural exudate cells (PLEC) were analysed, to gain insight on effects at sites distal from the site of injection of the stimuli. None of the treatments caused significant changes in the numbers of dead cells in the peritoneal or pleural cavity (Figure S1).

**Figure 1:**
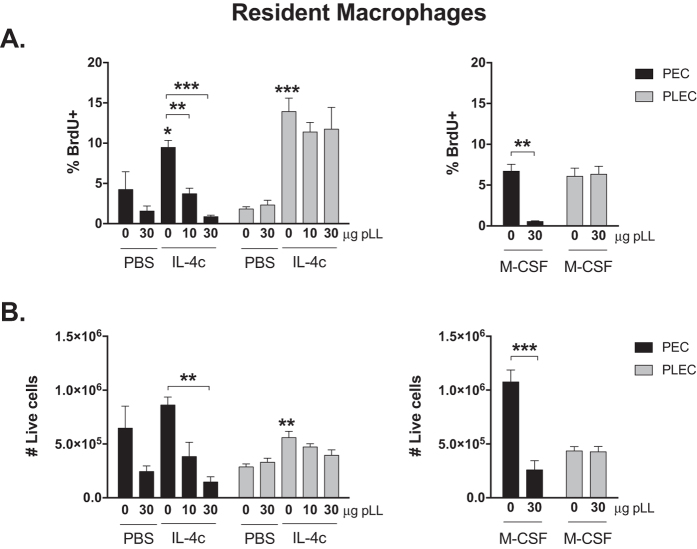
pLL inhibits IL-4- and M-CSF-induced proliferation of tissue resident macrophages *in vivo*. C57BL/6 mice were injected i.p. with IL-4c or M-CSF in combination with the indicated doses of pLL. A 3 hour pulse of BrdU was administered to all mice immediately before endpoint. Twenty-four hours post IL-4c/M-CSF injection, the percentage of these cells undergoing proliferation (%BrdU^+^) (**A**), as well as the total number of tissue resident macrophages (**B**) in the peritoneal (black bars) and pleural cavity (grey bars) was assessed by flow cytometry. Bars depict mean and SEM of 5 animals per group. The results are representative of two independent experiments. *p ≤ 0.05; **p ≤ 0.01; ***p ≤ 0.001 (asterisks not associated with connecting lines represent differences with respect to mice injected with PBS only).

As we chose an experimental system that employs a single dose of IL-4c or M-CSF-Fc, neither of which gives rise to robust increases in macrophage numbers within 24 hours, proliferation was analysed in terms of increases in the percentage of BrdU positive cells (%BrdU^+^). Resident peritoneal macrophages are F4/80^high^ (see gating strategy delineated in Figure S2)^33^. As expected, IL-4c induced the proliferation of this cell population, both in the peritoneal and pleural cavities (Fig. 1A), consistent with previous observation of systemic effects of IL-4c injected i.p.^4^. Injection of pLL in addition to IL-4c caused strong, dose-dependent inhibition in %BrdU^+^, in the peritoneal cavity but not the pleural cavity (Fig. 1A). Although the dose of M-CSF injected did not cause significant increases in BrdU^+^ resident macrophages with respect to PBS-injected animals, co-administration of pLL significantly decreased %BrdU^+^ cells with respect to M-CSF alone, also in the peritoneal cavity only (Fig. 1A). The inhibition of the proliferative effect of M-CSF probably explains a trend towards lower %BrdU^+^ resident macrophages in animals injected with pLL alone *vs* control animals (Fig. 1A), as M-CSF sustains macrophage proliferation under basal conditions^8^,^9^. Representative dot-plots of BrdU incorporation in resident peritoneal macrophages in mice injected IL-4c or M-CSF and/or 30 μg pLL are shown in Figure S3.

In addition to the effect on proliferation, the injection of pLL in combination with IL-4c or with M-CSF caused the disappearance of resident macrophages, compared to injection of IL-4c or M-CSF alone (Fig. 1B). Administration of pLL seemed to have a similar effect in the absence of IL-4c or M-CSF, although this was masked by the high deviations observed in the PBS only group. Again, the effects of pLL injection were local, i.e. pLL caused no significant decreases in macrophage numbers in the pleural cavity (Fig. 1B). The combination of disappearance of resident macrophages and proliferative inhibition resulted in 87% and 99% reductions in the numbers of resident peritoneal macrophages undergoing proliferation in response to IL-4c in mice injected with 10 and 30 μg pLL, respectively. Similarly, 30 μg pLL caused a 97% decrease in the number of resident macrophages proliferating in response to M-CSF.

### pLL inhibits proliferation of non-resident macrophages *in vivo*

**Figure 2:**
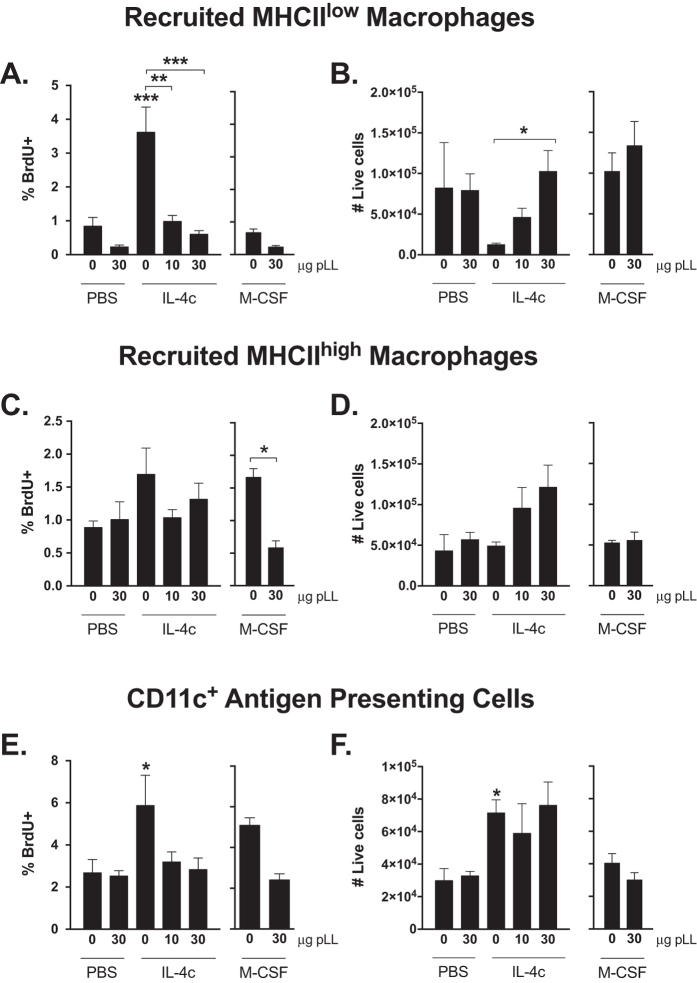
pLL inhibits IL-4 and M-CSF induced proliferation of non-tissue resident myeloid cells *in vivo.* C57BL/6 mice were treated as described in Fig. 1. Twenty-four hours post IL-4c/M-CSF injection the percentage of BrdU^+^ cells (**A**,**C**,**E**) and the total number (**B**,**D**,**F**), gated on recruited MHCII^low^ and MHCII^high^ macrophages and CD11c^+^ APCs, in the peritoneal cavity were assessed by flow cytometry. Bars depict mean and SEM of 5 animals per group. Results shown are representative of two independent experiments. *p ≤ 0.05; **p ≤ 0.01; ***p ≤ 0.001 (asterisks not associated with connecting lines represent differences with respect to mice injected with PBS only).

The mouse peritoneal cavity contains, in addition to F4/80^high^ resident macrophages, F4/80^low^ monocyte/macrophages recently recruited from circulation. Recruitment takes place constitutively, and is enhanced by inflammatory stimuli^33^,^34^. These cells can be divided into MHCII^low^ and MHCII^high^ subpopulations, the first one representing newly arrived cells that give rise to the second subpopulation^33^,^34^. We will call these subpopulations MHCII^low^ and MHCII^high^ recruited macrophages (Figure S2). Similar to its effect on resident macrophages, IL-4c induced the proliferation of such recruited macrophages in the peritoneal cavity (Fig. 2), an effect that reached significance for MHCII^low^ macrophages (Fig. 2A). Again similar to resident macrophages, the proliferation caused by IL-4c was also observable in the pleural cavity (Figure S4), reaching significance for MHCII^high^ macrophages (Figure S4C). We also analysed F4/80^low^ MHCII^high^ CD11c^+^ cells, a gate previously considered as corresponding to DCs only, but now recognised to additionally contain bone marrow-derived macrophages^7^,^34^. We will refer to cells in this gate as CD11c^+^ antigen-presenting cells (APC) (Figure S2). In this population, IL-4c also induced proliferation, both in the peritoneal (Fig. 2E) and pleural cavities (Figure S4E).

Similar to resident macrophages, administration of pLL together with IL-4c tended to decrease the %BrdU^+^ in the other three macrophage-containing gates in the peritoneal cavity (Fig. 2); this effect reached significance for MHCII^low^ recruited macrophages (Fig. 2A). Administration of pLL also tended to decrease the %BrdU^+^ in the context of co-administration of M-CSF, the effect reaching statistical significance for MHCII^high^ recruited macrophages (Fig. 2C). Representative dot-plots of BrdU incorporation in the non-resident peritoneal macrophage-containing populations in mice injected IL-4c or M-CSF and/or 30 μg pLL are shown in Figure S3. As for resident macrophages, the inhibition of non-resident macrophage proliferation caused by pLL was absent from the pleural cavity (Figure S4A,C,E).

At the doses used in this study, injection of pLL did not cause increases in recruited macrophage numbers in PEC in comparison to animals injected PBS alone (Fig. 2B,D,F). However, injection of pLL together with IL-4c tended to increase the number of recruited macrophages when compared to injection of IL-4c alone; this effect reached statistical significance for the MHCII^low^ subpopulation (Fig. 2B). Together with the disappearance of resident macrophages (Fig. 1B), this led to a shift in the proportion of resident *vs* recruited macrophages in the peritoneal cavity, from approximately 14:1 in mice administered IL-4c alone to 0.6:1 in mice given IL-4c plus pLL. This shift in CD11c^−^ macrophage subpopulations is depicted in Figure S5A,B. It is noteworthy that pLL injection at the doses used in the study, either as sole stimulus or in the context of co-injection with IL-4c or M-CSF, did not cause increases in total number of macrophages in the peritoneal cavity. In fact, it tended to cause decreases in total macrophage numbers, an effect that reached significance in the context of co-injection with M-CSF (Figure S5A). Along the same lines, in none of the conditions tested did pLL injection cause significant increases in total cell numbers in the peritoneal cavity (Figure S5C). With respect to the pleural cavity, injection of pLL as sole stimulus seemed to cause an increase in recruited MHCII^low^ macrophages (Figure S4B), but this effect was not reproduced in a repeat experiment. Other than this, no effects of pLL on the cell numbers in the non-resident macrophage-containing populations under study were observed in the pleural cavity (Figure S4B,D,F).

### pLL has differential effects on M(IL-4) markers *in vivo*

**Figure 3:**
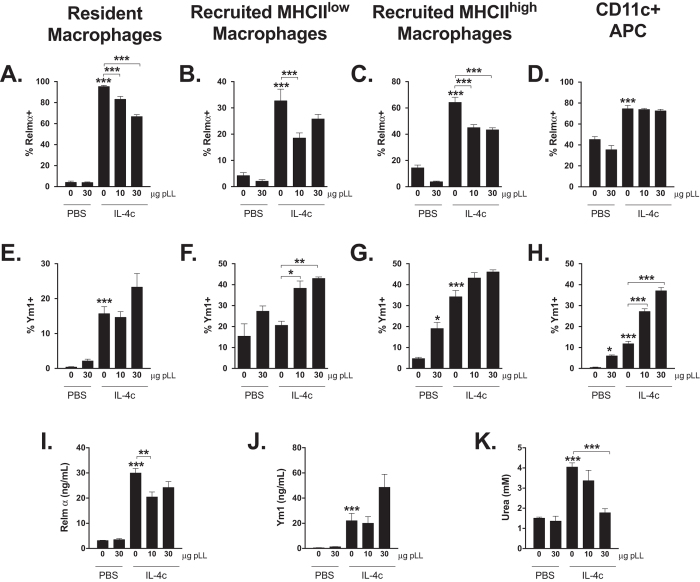
Injection of pLL has complex effects on M(IL-4) markers induced by co-injection of IL4-c. Mice were treated as described in Fig. 1. Twenty-four hours post injection the proportion of peritoneal cells expressing Relm-α (**A–D**) or Ym1 (**E–H**) was assessed by flow cytometry in the resident macrophage, recruited MHCII^low^ and MHCII^high^ macrophage, and CD11c^+^ APCs gates. Also, the concentration of secreted Relm-α (**I**) and Ym1 (**J**), as well as arginase activity in terms of urea formed in an *in vitro* reaction (**K**) were measured in the peritoneal lavage fluid. Bars depict mean and SEM of 5 animals per group. The results are representative of two independent experiments. *p ≤ 0.05; **p ≤ 0.01; ***p ≤ 0.001 (asterisks not associated with connecting lines represent differences with respect to mice injected with PBS only).

Injection of IL-4c also induced, as previously reported^4^,^5^,^35^, the expression of M(IL-4) markers Relm-α and Ym1 in the macrophage-containing cell gates under study in the peritoneal cavity, as well as increases in these two proteins and in Arginase activity in the corresponding lavage fluid (Fig. 3). Similar changes, except for the induction of soluble Arginase activity, were detected in pleural lavage fluid (Figure S6). Administration of pLL in combination with IL-4c caused a moderate but significant inhibition in Relm-α expression in resident and recruited (CD11c^−^) macrophages, which was accompanied by a similar effect on Relm-α levels in peritoneal lavage fluid (Fig. 3A–C,I). Arginase activity measured in the peritoneal lavage fluid was also inhibited by co-injection of pLL (Fig. 3K). In contrast, injection of pLL did not inhibit the expression of Ym1, but gave rise to a trend in the opposite direction (Fig. 3E,G,J), which reached statistical significance for the MHCII^low^ recruited macrophages and CD11c^+^ APC gates (Fig. 3F,H). In the absence of IL-4c administration, pLL at the doses tested did not *per se* induce any of the M(IL-4) markers tested, except for increases in the percentage of Ym1^+^ cells in the recruited MHCII^high^ macrophage and CD11c^+^ APC gates (Fig. 3G,H). In none of the experimental groups were significant levels of nitrite, TNF-α, IL-6 or IL-12 detected in the lavage fluids (data not shown). This indicates that the inhibition of Relm-α and Arginase upon injection of pLL was not due to classical activation of macrophages or DCs. In agreement, we have previously shown pLL to be free of endotoxins and not to induce, either *in vitro* or *in vivo*, any of several cytokines that are usually dependent on TLR triggering in dendritic cells and macrophages^31^. None of the effects of pLL on M(IL-4) markers were consistently detected in cells or fluid from the pleural cavity (Figure S6 and data not shown).

### pLL inhibits myeloid cell proliferation *in vitro*

**Figure 4:**
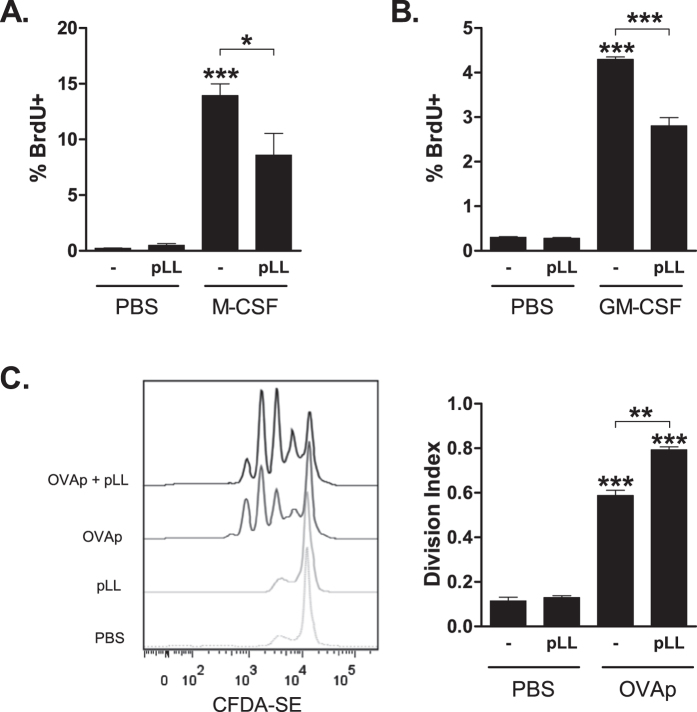
Exposure to pLL inhibits macrophage and BMDC proliferation *in vitro*. (**A**) Thioglycollate-elicited MΦ were stimulated *in vitro* with M-CSF in the presence or absence of 50 μg pLL (total dry mass) per million cells for 24 hours. A BrdU pulse was given 4 hours before endpoint and BrdU incorporation assessed by flow cytometry. (**B**) As A, using BMDC stimulated with GM-CSF in the presence or absence of 25 μg of pLL (total dry mass) per million cells. In (**A**,**B**), bars depict mean and SEM of %BrdU^+^ cells. (**C**) Proliferation of CFDA-SE labeled OT-II cells co-cultured with OVA peptide-primed BMDC in the presence or absence of 25 μg of pLL (total dry mass) per million BMDC. Both a representative histogram and quantitation of the division index for T cells are shown. The data shown are representative of two independent experiments. *p ≤ 0.05; **p ≤ 0.01; ***p ≤ 0.001 (asterisks not associated with connecting lines represent differences with respect to cells treated with medium only).

To gain insight into whether pLL inhibits proliferation by acting directly on myeloid cells, we tested its effects on thioglycollate-elicited macrophages (ThioMϕ) *in vitro.* We additionally tested GM-CSF bone marrow-derived dendritic cells (BMDCs), which are proposed to contain both DCs and CD11c^+^ macrophages^36^. For these experiments proliferation was induced with the known growth factors, M-CSF for ThioMϕ and GM-CSF for BMDCs. IL-4 was not used because it induces limited proliferation *in vitro*^32^. Both M-CSF and GM-CSF induced increases in %BrdU^+^ cells compared to the medium controls, and these increases were partially inhibited in the presence of pLL (Fig. 4A,B); representative dot-plots are shown in Figure S7. The inhibitory effects were not due to cell death, as there were no significant differences in cell viability between different experimental conditions (data not shown). There was no inhibitory effect of pLL on the antigen-specific proliferation of T cells as seen in BMDC-OTII co-cultures (Fig. 4C). This suggests that pLL does not cause a general inhibition of cell proliferation, but rather a myeloid cell-specific effect.

### Exposure to pLL inhibits IL-4-driven Relm-α expression *in vitro*

**Figure 5:**
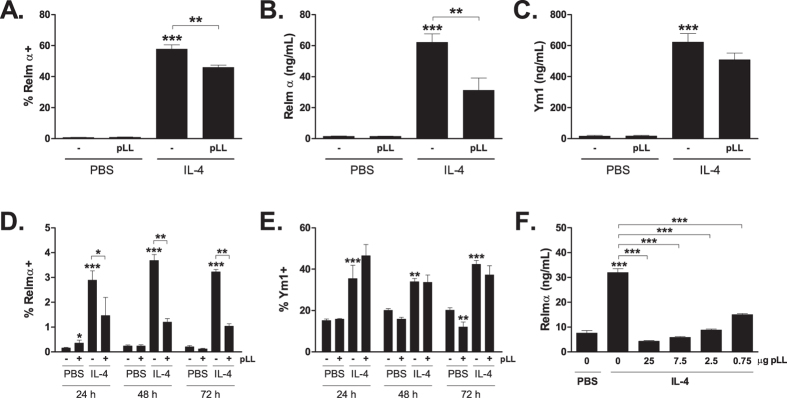
Exposure to pLL *in vitro* inhibits expression of Relm-α induced by IL-4 in macrophages and BMDCs. ThioMΦ were stimulated with IL-4 for 24 h in the presence or absence of pLL (50 μg per million cells). The percentage of cells expressing Relm-α was determined by flow cytometry (**A**), and the levels of Relm-α (**B**) and Ym1 (**C**) in supernatants was measured by ELISA. Also, BMDCs were stimulated with IL-4 and in the absence or presence of pLL (25 μg per million cells) for the indicated times, and then analysed by flow cytometry for expression of the M(IL-4) markers Relm α (**D**) and Ym1 (**E**). In addition, BMDCs were stimulated with IL-4 for 18 h in the absence or presence of different doses of pLL, and Relm-α was quantified in cell supernatants by ELISA (**F**). Statistically significantly differences are indicated: *p ≤ 0.05; **p ≤ 0.01; ***p ≤ 0.001 (asterisks not associated with connecting lines represent differences with respect to cells treated with medium only). In parts D and E, a two-way ANOVA was applied. The data are representative of at least two independent experiments; the diminution caused by pLL in the levels of Relm-α in the supernatants of BMDCs stimulated with IL-4 was variable, being in some experiments weaker than in the experiment shown in part (**F**).

In order to assess whether the effects on Relm-α and Ym1 expression observed *in vivo* could be reproduced *in vitro*, ThioMϕ or BMDCs were incubated with recombinant IL-4 in the presence or absence of pLL. As mentioned, BMDCs are proposed to contain CD11c^+^ macrophages and DCs^36^; DCs respond to IL-4 with an alternative activation program similar to macrophages^35^. The inhibitory effect of pLL on Relm-α was observed for ThioMϕ, which exhibited a slight but significant reduction of the IL-4-induced increase in intracellular Relm-α expression (Fig. 5A), more readily detectable in the amount of secreted Relm-α in the cell-culture supernatants (Fig. 5B). The amount of secreted Ym1 was not significantly altered (Fig. 5C). pLL had similar effects on BMDCs, with a strong reduction in the percentage of cells positive for Relm-α, without similar effect on Ym1 (Fig. 5D,E) or two other M(IL-4) markers, the mannose receptor (CD206) and PD-L2 (Figure S8). The inhibition of Relm-α secreted into cell supernatants by BMDCs could be detected even at very low doses of pLL (less than 1 μg per million cells) (Fig. 5F). The contrast between the effects of pLL on the IL-4-induced expression of Relm-α and Ym1 was also verified in bone marrow-derived macrophages (BMDMs), at the transcriptional level (Figure S9). Thus, both *in vitro* and *in vivo* the presence of pLL inhibits the IL-4-induced up-regulation of Relm-α but not that of Ym1.

### Exposure to pLL inhibits Akt phosphorylation *in vitro*

We hypothesised that inhibition of the PI3K/Akt pathway may be a prime candidate to explain the effects of pLL exposure on myeloid cells described thus far. The PI3K/Akt pathway is activated by IL-4 as well as by growth factors including M-CSF and GM-CSF, and it is necessary for cell proliferation^37^,^38^. Accordingly, the Akt inhibitor triciribine blocks IL-4c-induced macrophage proliferation *in vivo*^32^. In addition, the induction of Relm-α and Arginase depends on PI3K/Akt, in contrast to Ym1, which is PI3K/Akt-independent^32^,^39^.

**Figure 6:**
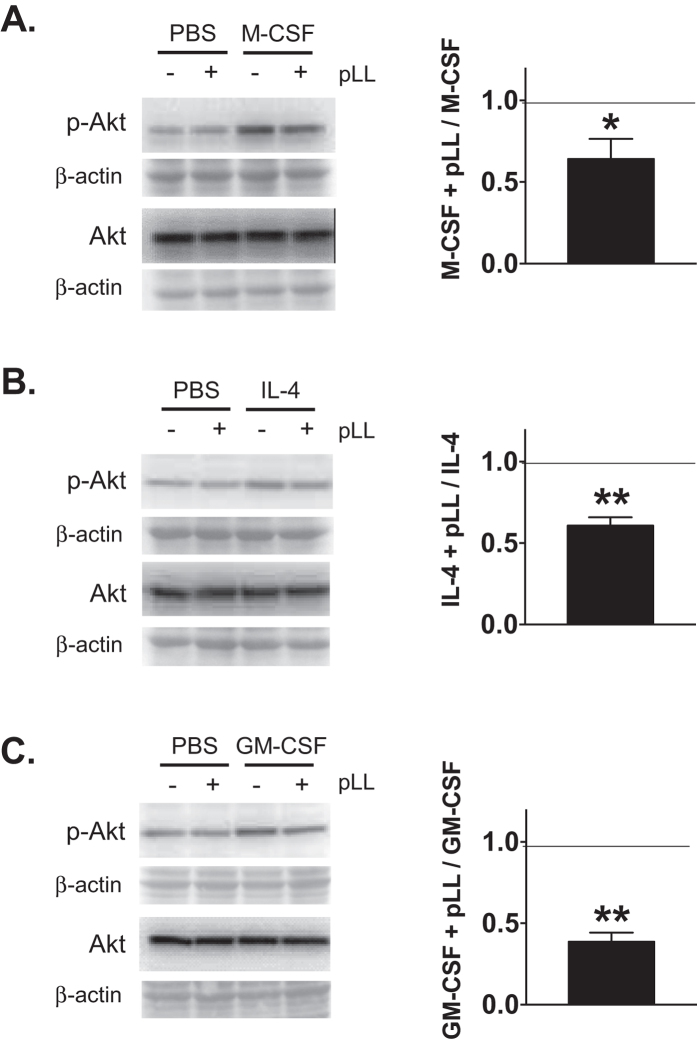
Exposure to pLL *in vitro* inhibits Akt phosphorylation in macrophages and BMDCs. ThioMΦ (**A**) or BMDC (**B**,**C**) were stimulated with M-CSF, IL-4 or GM-CSF as indicated, in the absence or presence of pLL (50 μg and 25 μg per million cells, respectively) and 80 minutes later cells were lysed for analysis of p-Akt and total Akt levels. Western blot results on the left are representative of four or five experiments (p-Akt) or two experiments (total Akt); the corresponding full-length blots are shown in Figure S11. Graphs on the right show the quotient of p-Akt values (normalised over Ponceau-stained β-actin loading controls; the identity of β-actin band was confirmed by mass spectrometry) for cells treated with pLL plus PI3K agonist over cells treated with PI3K agonist alone. Means and SEM for 4 or 5 independent experiments are plotted, and significance values by one-sample t test for the comparison with unity (i.e. no inhibition) in each case are given: *p ≤ 0.05; **p ≤ 0.01.

In agreement with the hypothesis above, incubation with pLL inhibited Akt phosphorylation in myeloid cells, without affecting total Akt levels. This was observed in ThioMϕ stimulated with M-CSF (Fig. 6A), and more strongly in BMDCs stimulated with either IL-4 or GM-CSF (Fig. 6B,C); we were not able to detect the expected increase in p-Akt in response to IL-4 in ThioMϕ (data not shown).

Growth factors are reported to cause a transient increase in ERK activation necessary for macrophage proliferation^40^. IL-4 is not known to signal through ERK^37^, and at least for macrophages it is only capable of robustly stimulating proliferation *in vivo*. TLR agonists induce prolonged ERK activation in macrophages, which inhibits, rather than induces, macrophage proliferation^40^. It was conceivable therefore that pLL acts, with respect to ERK, similarly to TLR agonists, thus inhibiting proliferation through excessive ERK activation. To test this possibility, ThioMϕ were incubated with pLL, M-CSF, or both, and levels of phosphorylated ERK (p-ERK) measured. pLL did not induce ERK phosphorylation *per se*, or significantly alter the phosphorylation induced by the growth factor (Figure S10). Therefore ERK does not seem to be involved in the inhibition of macrophage proliferation caused by pLL.

## Discussion

Macrophages proliferate locally in diverse models of immunological granuloma^41^,^42^,^43^,^44^,^45^ and in the local reaction against several different tissue-dwelling helminths^1^,^5^,^7^. Macrophage-rich, granulomatous reactions are associated with poor survival/vitality of *Echinococcus* larvae, but these reactions do not develop in most host species/contexts^15^,^16^,^17^,^18^,^19^,^23^,^24^. Thus we reason that the parasite may be adapted to avoid macrophage accumulation and granuloma formation through inhibition of proliferative expansion. Our data suggests that this may be the case, and that proliferation is inhibited at least in part through properties of the LL, the only structure with which host cells normally make contact^15^,^25^,^26^. In other words, macrophages making contact with the LL or with particles derived from it may receive an anti-proliferative signal contributing to the inflammatory control most usually observed in hydatid infection.

Our findings suggest that LL materials may be able to inhibit macrophage proliferation irrespective of the extracellular proliferative signal involved. This is potentially important, because IL-4 and M-CSF can cooperate to drive macrophage proliferation in inflammatory Th2 settings, and additional cytokines are proposed to induce proliferation in granulomatous inflammation in particular^1^,^5^,^7^,^44^. Of note, our data indicates that pLL- mediated inhibition of proliferation is restricted to the site of injection (Figs 1 and S5). Thus inflammatory control by LL particles is likely limited to the area surrounding the hydatid, thus retaining host fitness in the face of other infections.

Depending on dose, pLL can cause macrophage recruitment (Fig. 2A; data not shown). It is hard to know whether LL particles released slowly from a hydatid in the infection context would have the same pro-recruitment effect. In any case, further proliferative expansion of these recruited cells, as found in other inflammatory settings^7^, would be inhibited, as suggested by the observation that injection of pLL reduced proliferation of recruited macrophages in response to IL-4c (Fig. 2).

Particulate inflammatory stimuli generally cause disappearance of resident macrophages and recruitment of macrophages of bone marrow origin^46^,^47^,^48^,^49^,^50^,^51^, two phenomena that we will refer to together as “disappearance-recruitment”. pLL, especially at high doses, causes “disappearance-recruitment” (Figs 1B, 2B and S3A,B; data not shown), indicative of potential to elicit acute inflammation. However, we reason that inflammation is not the cause of the inhibitory effect on macrophage proliferation, for the reasons that follow. Published studies using zymosan show that acute inflammatory stimuli actually promote proliferation of both resident and recruited macrophages^47^,^48^. In possible coincidence, doses of pLL higher than those used in this study cause stronger disappearance-recruitment but *weaker* inhibition of IL-4-driven proliferation (data not shown). Finally, we observed inhibition of proliferation also *in vitro*, in two different models (Fig. 4A,B).

The most likely mechanistic explanation for the effect of pLL on macrophage proliferation is that contact with the material inhibits activation of the PI3K effector Akt (Fig. 6). The PI3K/Akt pathway is central for cell survival in all cell types, and recent data implicate PI3K and/or Akt in proliferation of macrophages and other myeloid cells in response to M-CSF and to IL-4c *in vivo*^32^,^52^,^53^,^54^,^55^. We have recently found that pLL suppresses Akt phosphorylation in response to LPS (Á. Pittini, unpublished), as seen here for IL-4 and GM-CSF. The impaired Akt phosphorylation in response to disparate stimuli strongly suggests that inhibition is independent of the receptor that triggers PI3K activation, which would explain inhibition of proliferation in response to different stimuli. The impaired Akt phosphorylation observed in all likelihood results in inhibited downstream signalling, as is deduced from the blunted up-regulation of M(IL-4) markers known to be PI3K/Akt dependent (Relm-α, Arginase) without similar effect on Ym1, known to be PI3K/Akt-independent (Figs 3,5, and S6)^32^,^39^,^56^,^57^,^58^. These results also suggest strongly that pLL does not interfere with the STAT6 pathway, globally necessary for induction of M(IL-4) markers and operating in this context in parallel to the PI3K/Akt pathway^37^,^39^.

The expected effect in macrophages/DCs of interaction with particles is activation of PI3K and Akt, which normally takes place within the first few minutes^59^,^60^,^61^,^62^. We cannot rule out that an initial phase of Akt activation occurs upon interaction with pLL, but in any case we consistently observe that the interaction makes cells refractory to concomitantly added PI3K agonists in terms of Akt activation (Fig. 6; unpublished data). Perhaps related to PI3K/Akt activation, a wide range of particulate preparations have been reported to stimulate, rather than inhibit, macrophage proliferation *in vitro*^63^,^64^,^65^,^66^. Therefore the interaction of pLL with macrophages appears to be unusual both in terms of signalling and impact on proliferation.

Injection of low doses of pLL enhanced the induction of Chil3/Ym1 by IL-4 (Fig. 3F,H) or even induced the marker by itself (Fig. 3G,H) in the different macrophage-containing gates. A higher dose of pLL (150 μg) alone induces in resident macrophages the expression of M(IL-4) markers, including Relm-α (data not shown). Therefore the effects of pLL on M(IL-4) markers *in vivo* probably reflect the superimposition of its capacity to inhibit PI3K/Akt (and thus Relm-α and Arginase expression) and a separate capacity to promote M(IL-4) activation. Other types of particles are known to induce the M(IL-4) phenotype *in vivo*^67^. Therefore the situation may be similar to what was discussed above: at high doses of pLL, effects that are expected of particles in general predominate (recruitment of monocyte-derived macrophages, disappearance of resident macrophages, induction of M(IL-4) markers) but at low doses, effects that may be unique of this material become apparent (inhibition of proliferation and of induction of the PI3K-dependent subset of M(IL-4) markers). We propose that these low-dose effects, on which the present paper is focused, reflect evolutionary adaptations to minimise the inflammatory potential of the LL and materials shed from it.

We have no direct information on the molecular interactions between pLL and myeloid cells that initiate the unusual effects on myeloid cells observed. It is unlikely that a specific carbohydrate - lectin receptor interaction is involved, because: (i) the triggering of unconventional maturation does not require intact glycans in pLL^31^ and (ii) none of 35 recombinant human innate immune receptors with carbohydrate-binding domains bound the LL in a receptor profiling study^68^. As we have pointed out previously^15^ the LL carbohydrates are structurally unrelated to the dominant carbohydrates in the larval stages of related *Taenia* parasites, known to condition the innate immune system through carbohydrate-host lectin interactions^69^,^70^. The nature of the interaction between the LL and myeloid cells, as well as the intracellular changes leading to altered Akt activation, are the subject of current studies. Beyond its relevance to cystic echinococcosis, our work identifies a material that upon interaction with myeloid cells elicits certain biological effects that are opposite to those expected of particles in general, possibly because of its also unusual capacity to inhibit PI3K/Akt activation.

## Methods

### Preparation of pLL

pLL was prepared from *E. granulosus* hydatids from naturally infected Uruguayan cattle, as described in^31^. Briefly, hydatid walls were thoroughly washed with 2 M NaCl, before being dehydrated by freeze-drying. The material was then finely ground and carefully rehydrated in endotoxin-free buffer. The resulting suspensions were sequentially filtered through 85- and 23- μm gauze, and again extensively washed in endotoxin-free PBS. Penicillin and streptomycin were added to the final suspensions, which were kept at 4 °C until used. pLL preparations tested negative for endotoxin by the *Limulus* amebocyte lysate (LAL) method^31^. Doses of pLL are expressed in terms of total dry mass, determined by washing the suspension into water, freeze drying, and weighing.

### Mice and *in vivo* experiments

*In vivo* experiments were performed in a specific pathogen-free facility at the University of Edinburgh, using C57BL/6 female mice of 8–12 weeks of age. Mice were injected intraperitoneally (i.p.) with 1 μg recombinant mouse IL-4 (PeproTech) complexed to 5 μg anti-IL-4 antibody (clone 11B11; BioXCell); this complex, which has a molar ration of 2:1, is termed IL-4c^4^. Alternatively, mice were injected with 5 μg of the fusion protein comprising mouse M-CSF and the Fc portion of pig IgG, kindly provided by Prof. D. Hume (University of Edinburgh)^5^. Injection of PBS was used as control in all cases. Where indicated, mice were subsequently injected i.p. with different doses of pLL (10 or 30 μg per mouse). Twenty-one hours post IL-4c/M-CSF injection, mice received 100 μL of 10 mg/mL 5-Bromo-2′-deoxyuridine (BrdU; Sigma Aldrich) subcutaneously. Three hours later, animals were sacrificed in a CO_2_ chamber and peritoneal exudate cells (PEC) and pleural exudate cells (PLEC) were harvested in RPMI 1640 containing penicillin/streptomycin; in addition, peritoneal and pleural fluids were was collected.

### Thioglycollate-elicited macrophages (ThioMΦ)

For *in vitro* experiments, macrophages were elicited by i.p. injection of 400 μL 4% w/v aged Brewer’s modified thioglycollate medium (BD Biosciences). Three days post-injection, PEC were harvested and seeded into 96-well plates at 2 × 10^5^ cells per well in RPMI 1640 containing 5% v/v FCS and penicillin/streptomycin. Non-adherent cells were washed off after 4 hours incubation.

### BMDC cells and macrophages

BMDC were generated by the method of Lutz^71^, as described in^31^. Bone marrow-derived macrophages (BMDM) were prepared from bone marrow precursors in the presence of conditioned medium from the L929 cell line, containing M-CSF, as described in^72^.

### *In vitro* proliferation assay

ThioMΦ were stimulated with 20 ng/mL recombinant mouse M-CSF (PeproTech) in the presence or absence of pLL (50 μg per million cells). BMDC were stimulated with 20 ng/mL recombinant mouse GM-CSF (PeproTech) in the presence or absence of pLL (25 μg pLL per million cells). Twenty hours post-stimulation, a pulse of 10 μM BrdU was administered. Four hours later, cells were placed on ice and stained for flow cytometry.

### BMDC – OT-II co-culture

CD4^+^ T lymphocytes from B6.Cg-Tg(TcraTcrb)425Cbn/J transgenic mice (known as OT-II mice), which are specific for the 323–339 peptide of ovalbumin (OVA), were purified from splenic and lymphatic nodes cells using the Dynal Mouse CD4 Cell Negative Isolation Kit (Invitrogen) and stained with 5 μM CFDA-SE (Thermo Scientific). BMDCs were seeded into 96-well round-bottomed plates (40 × 10^3^ per well), in the presence or absence of 1 μg pLL. After letting the BMDC settle for one hour, 200 × 10^3^ labelled T cells were added, in the presence or absence of 0.1 μg/mL OVA peptide. After three days of incubation, the co-culture was placed on ice and stained for flow cytometry.

### Flow cytometry

PEC or PLEC samples were treated with red blood cell lysis buffer (Sigma), counted, and equal numbers of cells were seeded for staining. All antibodies were purchased from BioLegend UK, unless stated otherwise. Cells, from *in vitro* or *in vivo* experiments, were stained with LIVE/DEAD-Far Red or -Aqua (Life Technologies) and then blocked with 5 μg/mL anti-CD16/32 (2.4G2; produced in-house) and heat-inactivated normal mouse serum (1:10) in FACS buffer (0.5% BSA and 2 mM EDTA in Dulbecco´s PBS). Surface staining was performed on ice with the following fluorochrome-conjugated antibodies: F4/80 (BM8), CD11c (N418), CD19 (6D5), I-A/I-E (M5/114.15.2), Ly6G (1A8), SiglecF (E50–2440, BD), TCRβ (H57–597). For intracellular staining, cells were then fixed with 2% paraformaldehyde (Sigma Aldrich) and permeabilised using Permeabilizing Solution (eBioscience). Cells were then stained with purified polyclonal rabbit anti-Relm-α (PeproTech) followed by an anti-rabbit IgG reagent, either purchased in fluorochrome-conjugated form (BioLegend) or labeled with the Zenon^TM^ labeling kit (Invitrogen). Alternatively, cells were stained with biotinylated goat anti-Ym1 (RnD Systems) followed by Streptavidin-PerCP. For intranuclear staining, cells were fixed with Foxp3/Transcription Factor Fixation/Permeabilization buffer (eBioscience) after surface staining. The cells were further treated with 100 μL of 765 KU/mL DNAse (Sigma) before staining with fluorochrome-conjugated anti-BrdU. Expression of Ym1, Relm-α and BrdU was determined relative to appropriate monoclonal or polyclonal isotype controls, or to non-DNAse treated samples, respectively. For *in vitro* T-cell proliferation assays, CSFE fluorescence was measured in cells gated for CD4 expression. Data were acquired using a BD CANTO II flow cytometer and analysed with Flowjo (version 7.6).

### ELISA, Arginase assay and Griess Assay

Cytokines were detected in peritoneal lavage fluid using commercial paired antibody kits: IL-12p40 (BD), IL-6 (BD), TNFα (R&D), Relm-α (PeproTech) and Ym1 (RnD). Arginase activity was measured in terms of urea produced from the hydrolysis of exogenous L-arginine added to the lavage fluid, as described elsewhere^73^. Nitrites as an indication of NO production were determined in lavage fluid by the Griess assay. For this purpose, equal volumes of sample and Griess reagent (4.93% w/v H_3_PO_4_, 58 mM sulfanilamide and 3.8 mM napthylene) were mixed, and absorbance was measured after 5 minutes at 540 nm.

### Western blotting

Cells were lysed with 1% m/v Triton X-100 in PBS containing phosphatase and protease inhibitors (Santa Cruz Biotechnology), and kept at -20 °C until blotting. Lysates were reduced/denatured, run on 10% acrylamide SDS-PAGE gels, and transferred to PVDF membranes (Millipore). The membranes were stained with Ponceau Red to obtain the loading controls, then washed, blocked with 0.1% m/v Tween-20, 0.2% m/v BSA in PBS, and probed with either anti-phospho-Akt (S473) (CST; 1/1000) or anti-phopho-ERK (T202/Y204) (CST; 1/2000) in blocking solution. After washing, the membranes were probed with HRP-conjugated anti-rabbit IgG (Calbiochem; 1/5000 in blocking solution) and developed with SuperSignal West Pico chemiluminescent substrate (Thermo Scientific). Quantification of the intensity of the bands was done with the ImageJ software.

### Quantitative PCR

RNA was recovered from cells by resuspension in TRIzol reagent (Invitrogen). Total RNA was extracted according to the manufacturer’s instructions. One μg of RNA was used for the synthesis of cDNA using Moloney murine leukemia virus reverse transcriptase. Relative quantification of genes was carried out by quantitative PCR using the Roche Lightcycler as described in^74^. Five serial 1/4 dilutions of a positive control sample of cDNA were used as a standard curve in each reaction, and the expression levels were estimated from the curve. Real-time PCR of the housekeeping gene HPRT allowed normalisation of the expression of the genes of interest. Primer sequences were as follows: HPRT-forward TCCTCCTCAGACCGCTTTT; HPRT-reverse CCTGGTTCATCATCGCTAATC; Ym1- forward TCACAGGTCTGGCAATTCTTCTG; Ym1-reverse TTGTCCTTAGGAGGGCTTCCTC; Relm-α-forward TATGAACAGATGGGCCTCCT; Relm-α-reverse GGCAGTTGCAAGTATCTCCAC.

### Ethics Statement

All animal experiments were performed in accordance with the UK Animals (Scientific Procedures) Act of 1986 under a Project License (60/4104) granted by the UK Home Office and approved by the University of Edinburgh Ethical Review Committee.

### Statistical Analyses

Statistical analyses were carried out with GraphPad Prism (version 5), by One-Way ANOVA with Tukey´s post-test unless stated otherwise. Differences were considered significant when p ≤ 0.05.

## Additional Information

**How to cite this article**: Seoane, P. I. *et al*. Particles from the *Echinococcus granulosus* laminated layer inhibit IL-4 and growth factor-driven Akt phosphorylation and proliferative responses in macrophages. *Sci. Rep.* **6**, 39204; doi: 10.1038/srep39204 (2016).

**Publisher’s note:** Springer Nature remains neutral with regard to jurisdictional claims in published maps and institutional affiliations.

## Supplementary Material

Supplementary Figures
