# Supplementary material for: Particles from the Echinococcus granulosus laminated layer inhibit IL-4 and growth factor-driven Akt phosphorylation and proliferative responses in macrophages
Source: Sci Rep. 2016 Dec 14;6:39204. doi: 10.1038/srep39204 (PMC5155279; doi:10.1038/srep39204)
Supplement: Supplementary Figures [file srep39204-s1.pdf]

Particles from the *Echinococcus granulosus* laminated layer inhibit IL-4 and growth factor-driven Akt phosphorylation and proliferative responses in macrophages

Paula I. Seoane, Dominik Rückerl, Cecilia Casaravilla, Anabella Barrios, Alvaro Pittini, Andrew S. MacDonald, Judith E. Allen & Alvaro Díaz

Supplemental Materials

Figure S1

A.

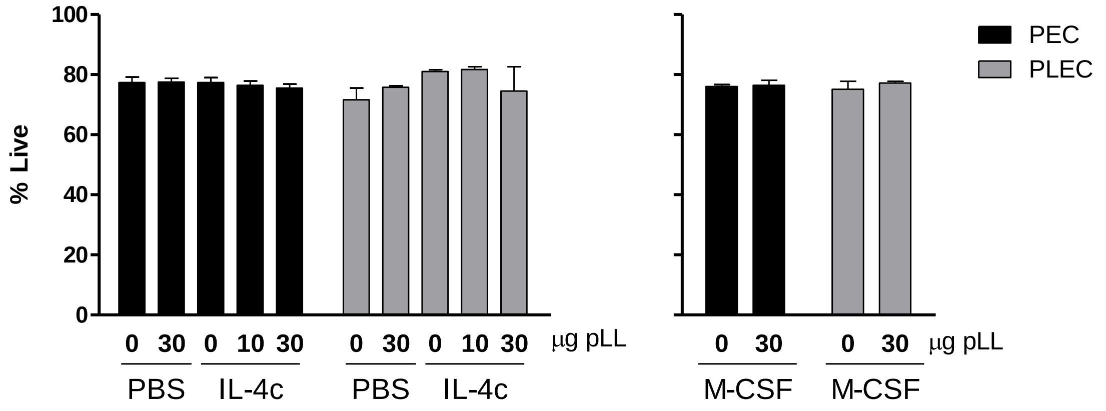

B.

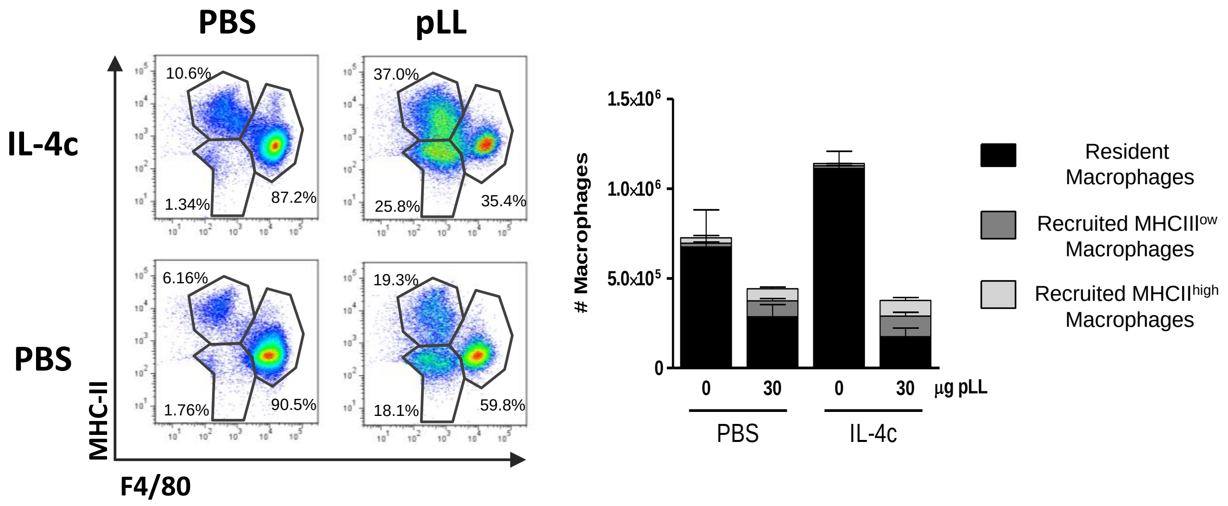

C.

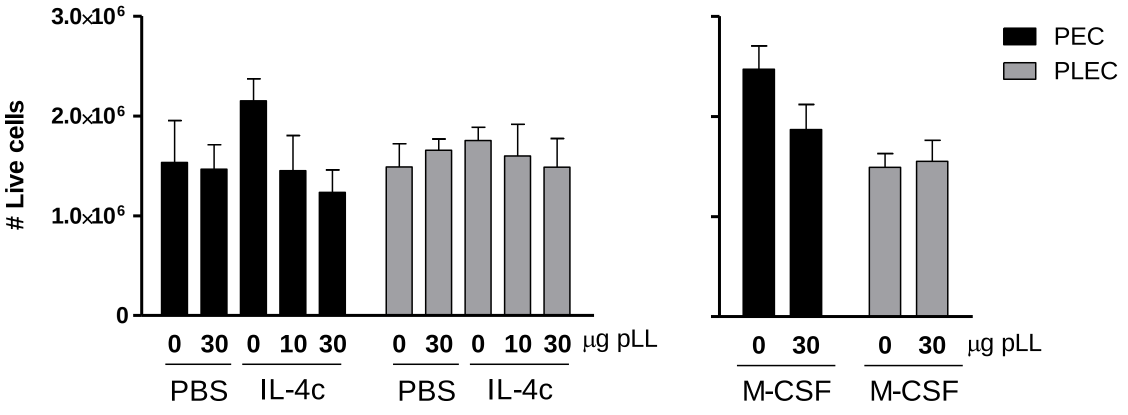

Figure S1. Injection of pLL up to 30 μg per mouse does not cause significant cell death. C57BL/6 mice were injected i.p. with IL-4c or M-CSF in combination with the indicated doses of pLL, and peritoneal lavage exudate (PEC) and pleural lavage exudate (PLEC) cells analysed for viability 24 hours later. Bars depict mean and SEM of 5 animals per group and are representative of two independent experiments.

Figure S2

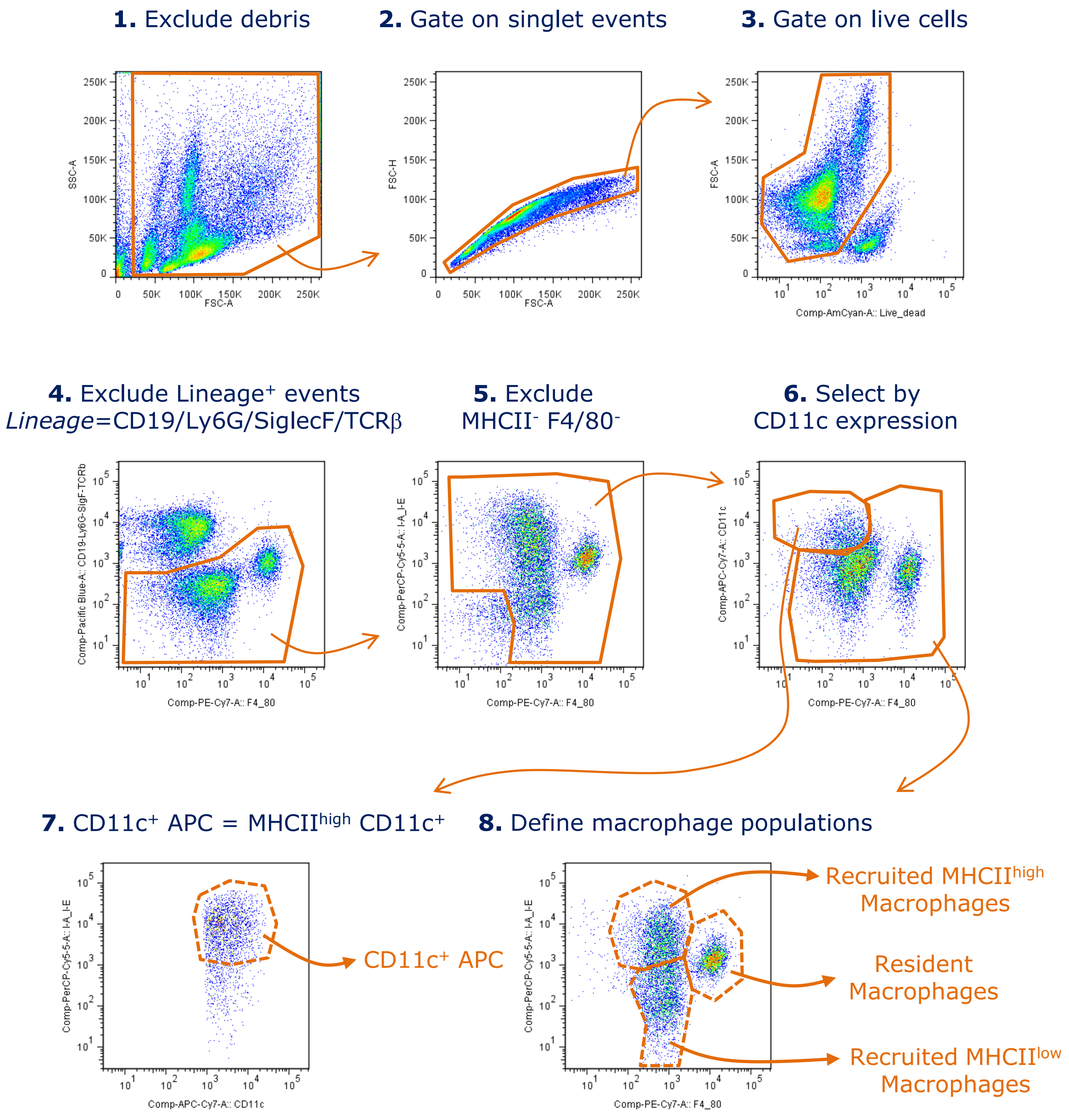

1. Exclude debris
2. Gate on singlet events
3. Gate on live cells
SSC-A
FSC-A
FSC-H
FSC-A
Comp-AmCyan-A:: Live_dead
4. Exclude Lineage+ events
Lineage=CD19/Ly6G/SiglecF/TCRβ
5. Exclude MHCII- F4/80-
6. Select by CD11c expression
Comp-Pacific Blue-A:: CD19-Ly6G-SigF-TCRb
Comp-PE-Cy7-A:: F4_80
Comp-PerCP-Cy5-5-A:: I-A_I-E
Comp-PE-Cy7-A:: F4_80
Comp-APC-Cy7-A:: CD11c
Comp-PE-Cy7-A:: F4_80
7. CD11c+ APC = MHCIIhigh CD11c+
8. Define macrophage populations
Comp-PerCP-Cy5-5-A:: I-A_I-E
Comp-APC-Cy7-A:: CD11c
CD11c+ APC
Comp-PerCP-Cy5-5-A:: I-A_I-E
Comp-PE-Cy7-A:: F4_80
Recruited MHCIIhigh Macrophages
Resident Macrophages
Recruited MHCIIlow Macrophages

Figure S2. Gating strategy for peritoneal and pleural cells. Four specific cellular populations were defined as follows. Resident Macrophages: (Live/Dead$^-$ CD19$^-$ Ly6G$^-$ SiglecF- TCRβ$^-$) CD11c$^-$ F4/80^{high}. Recruited MHCII^{high} macrophages: (Live/Dead$^-$ CD19$^-$ Ly6G$^-$ SiglecF$^-$ TCRβ$^-$) CD11c$^-$ F4/80^{low} MHCII^{high}. Recruited MHCII^{low} macrophages: (Live/Dead$^-$ CD19$^-$ Ly6G$^-$ SiglecF$^-$ TCRβ$^-$) CD11c$^-$ F4/80^{low} MHCII^{low}. CD11c$^+$ APC: (Live/Dead$^-$ CD19$^-$ Ly6G$^-$ SiglecF$^-$ TCRβ$^-$) CD11c$^+$ MHCII^{high}.

Figure S3

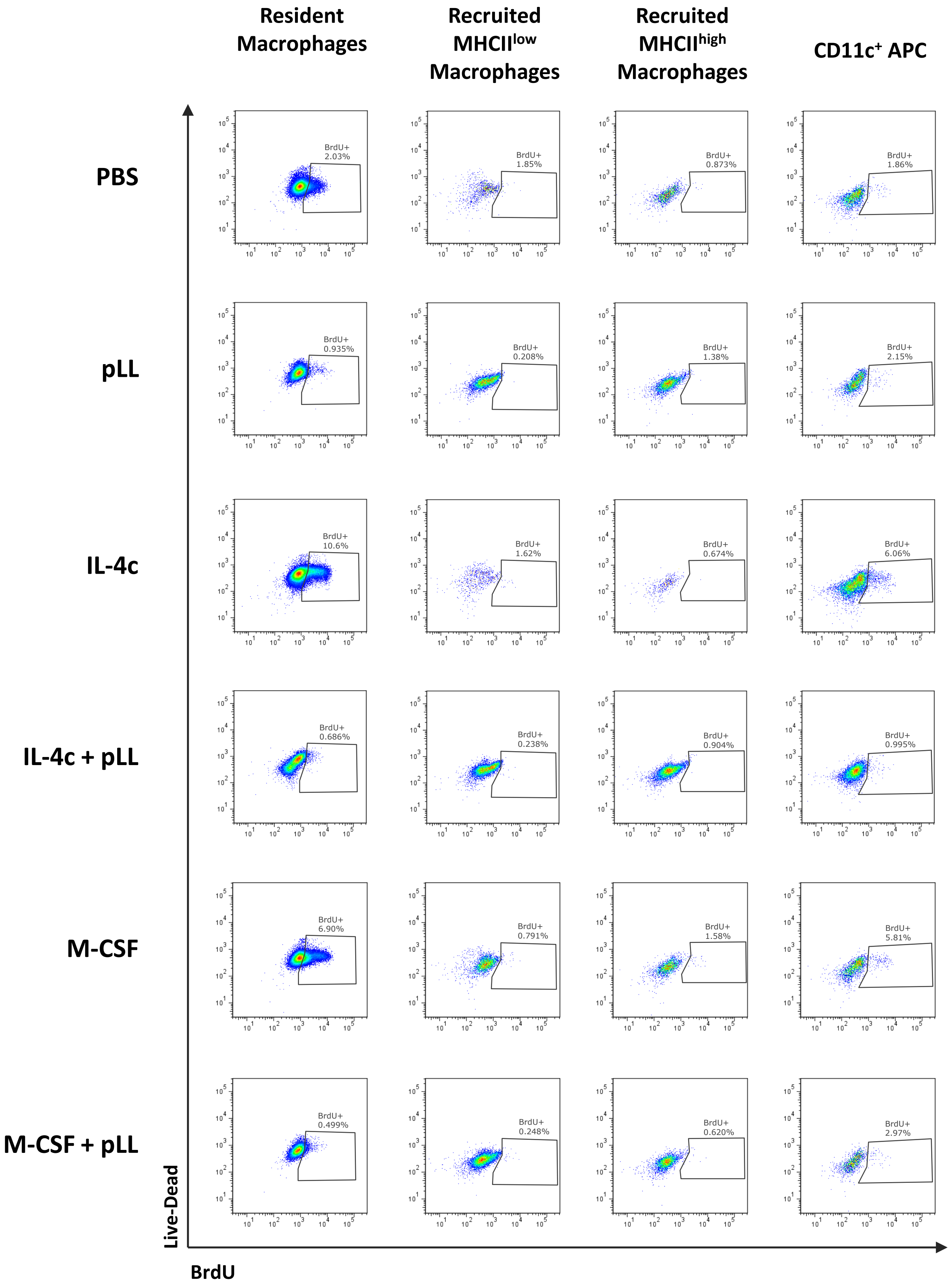
Resident Macrophages
Recruited MHCII^low Macrophages
Recruited MHCII^high Macrophages
CD11c+ APC
PBS
pLL
IL-4c
IL-4c + pLL
M-CSF
M-CSF + pLL
BrdU+ 2.03%
BrdU+ 1.85%
BrdU+ 0.873%
BrdU+ 1.86%
BrdU+ 0.935%
BrdU+ 0.208%
BrdU+ 1.38%
BrdU+ 2.15%
BrdU+ 10.6%
BrdU+ 1.62%
BrdU+ 0.674%
BrdU+ 6.06%
BrdU+ 0.686%
BrdU+ 0.238%
BrdU+ 0.904%
BrdU+ 0.995%
BrdU+ 6.90%
BrdU+ 0.791%
BrdU+ 1.58%
BrdU+ 5.81%
BrdU+ 0.499%
BrdU+ 0.248%
BrdU+ 0.620%
BrdU+ 2.97%
Live-Dead
BrdU

Figure S3. Representative dot-plots showing BrdU incorporation in four macrophage-containing peritoneal populations of mice injected IL-4c or M-CSF +/- pLL (30 μg). The results correspond to representative mice of the experiment shown in Figures 1 and 2.

Figure S4

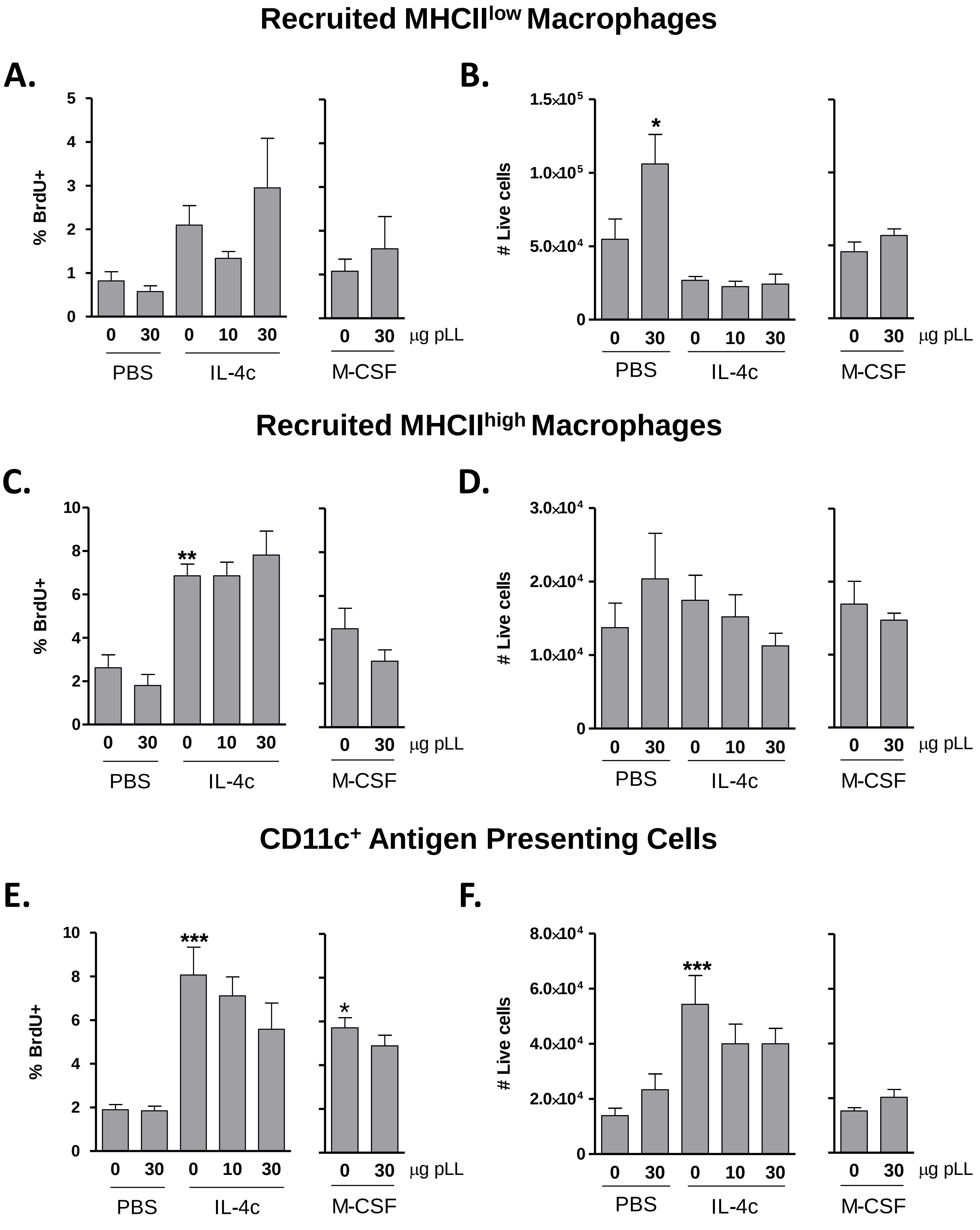
Recruited MHCII^low Macrophages
A.
% BrdU+
0 30 0 10 30
0 30 μg pLL
PBS
IL-4c
M-CSF
B.
Live cells
1.5x10^5
1.0x10^5
5.0x10^4
*
0 30 0 10 30
0 30 μg pLL
PBS
IL-4c
M-CSF
Recruited MHCII^high Macrophages
C.
% BrdU+
**
0 30 0 10 30
0 30 μg pLL
PBS
IL-4c
M-CSF
D.
Live cells
3.0x10^4
2.0x10^4
1.0x10^4
0 30 0 10 30
0 30 μg pLL
PBS
IL-4c
M-CSF
CD11c+ Antigen Presenting Cells
E.
% BrdU+
***
*
0 30 0 10 30
0 30 μg pLL
PBS
IL-4c
M-CSF
F.
Live cells
8.0x10^4
6.0x10^4
4.0x10^4
2.0x10^4
***
0 30 0 10 30
0 30 μg pLL
PBS
IL-4c
M-CSF

Figure S4. Peritoneal injection of pLL has no effect on proliferation of non-resident pleural myeloid cells. C57BL/6 mice were treated as described in Figure 1. Twenty-four hours post IL-4c/M-CSF injection the percentage of $BrdU^{+}$ cells (**A**, **C**, **E**) and the cell numbers (**B**, **D**, **F**), gated on recruited MHC^{low} and $MHCII^{high}$ macrophages and on $CD11c^{+}$ APCs in the pleural cavity were assessed by flow cytometry. Bars depict mean and SEM of 5 animals per group and are representative of two independent experiments (except that the increase in recruited $MHCII^{low}$ macrophage numbers caused by 30 µg pLL was not reproducible). * $p \leq 0.05$; ** $p \leq 0.01$; *** $p \leq 0.001$ (asterisks not associated with connecting lines represent differences with respect to cells treated with medium only).

Figure S5

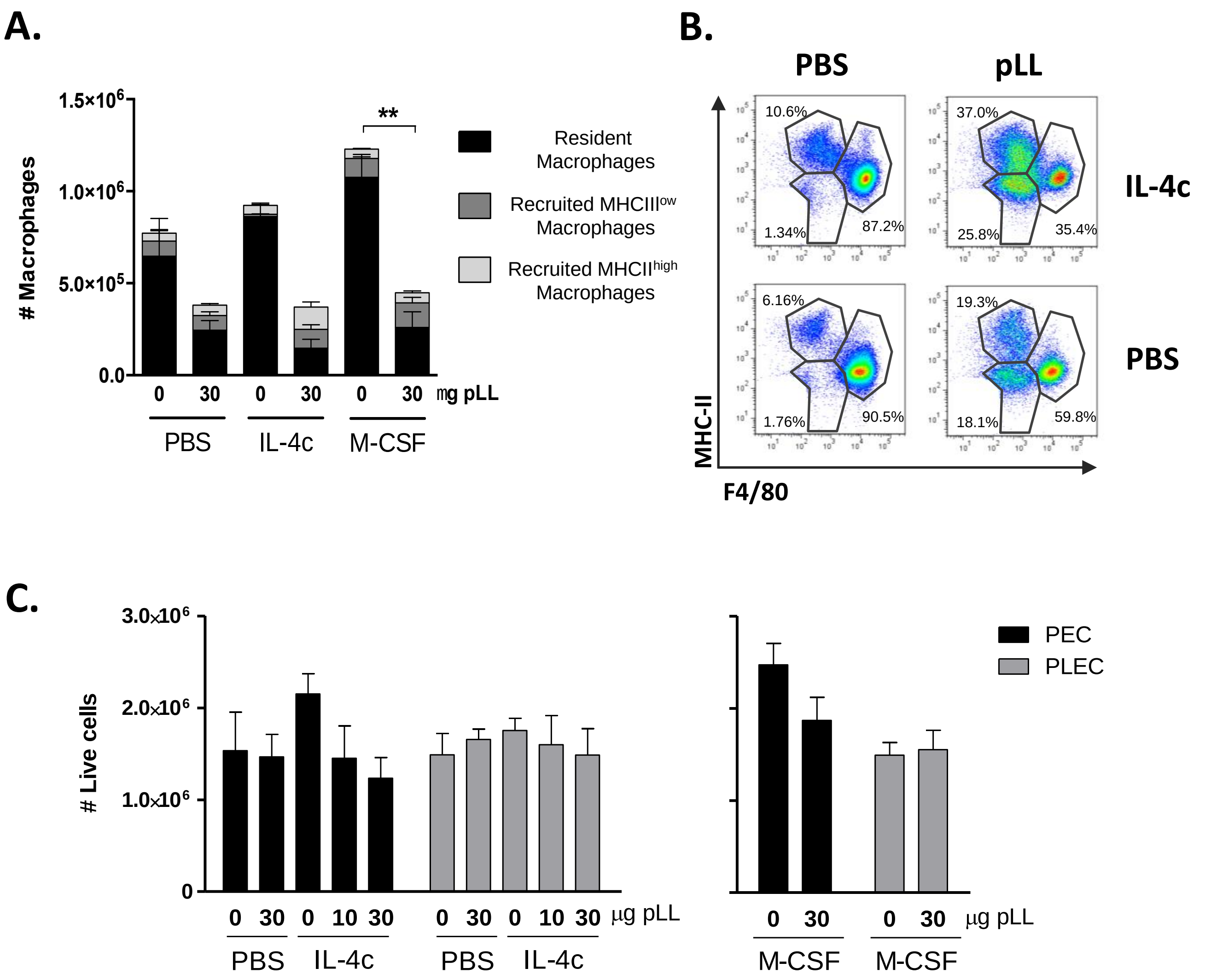

A.
1.5×10^6
1.0×10^6
5.0×10^5
0.0
Macrophages
**
Resident Macrophages
Recruited MHCIIlow Macrophages
Recruited MHCIIhigh Macrophages
0 30 0 30 0 30 mg pLL
PBS
IL-4c
M-CSF
B.
PBS
pLL
10.6%
87.2%
1.34%
37.0%
25.8%
35.4%
IL-4c
6.16%
1.76%
90.5%
19.3%
18.1%
59.8%
PBS
MHC-II
F4/80
C.
3.0x10^6
2.0x10^6
1.0x10^6
0
Live cells
0 30 0 10 30 0 30 0 10 30 µg pLL
PBS IL-4c PBS IL-4c
PEC
PLEC
0 30 0 30 µg pLL
M-CSF M-CSF

Figure S5. Injection of pLL causes shifts in macrophage subpopulations without increasing total macrophage or total cell numbers. C57BL/6 mice were injected i.p. with IL-4c or M-CSF in combination with the indicated doses of pLL, and peritoneal lavage exudate (PEC) and pleural lavage exudate (PLEC) cells analysed 24 hours later. **(A)** Numbers of resident, recruited MHCII^{low} and recruited MHCII^{high} macrophages, and of the sum of these macrophage gates in the peritoneal cavity. **(B)** Representative dot-plots of the three peritoneal cavity macrophage populations for which cell numbers are plotted in part (A), for mice injected IL-4c +/- 30 μg pLL; the populations are defined as shown in Figure S2. **(C)** Numbers of total cells in the peritoneal and pleural cavities. Bars depict mean and SEM of 5 animals per group and are representative of two independent experiments. ** $p \leq 0.01$.

Figure S6

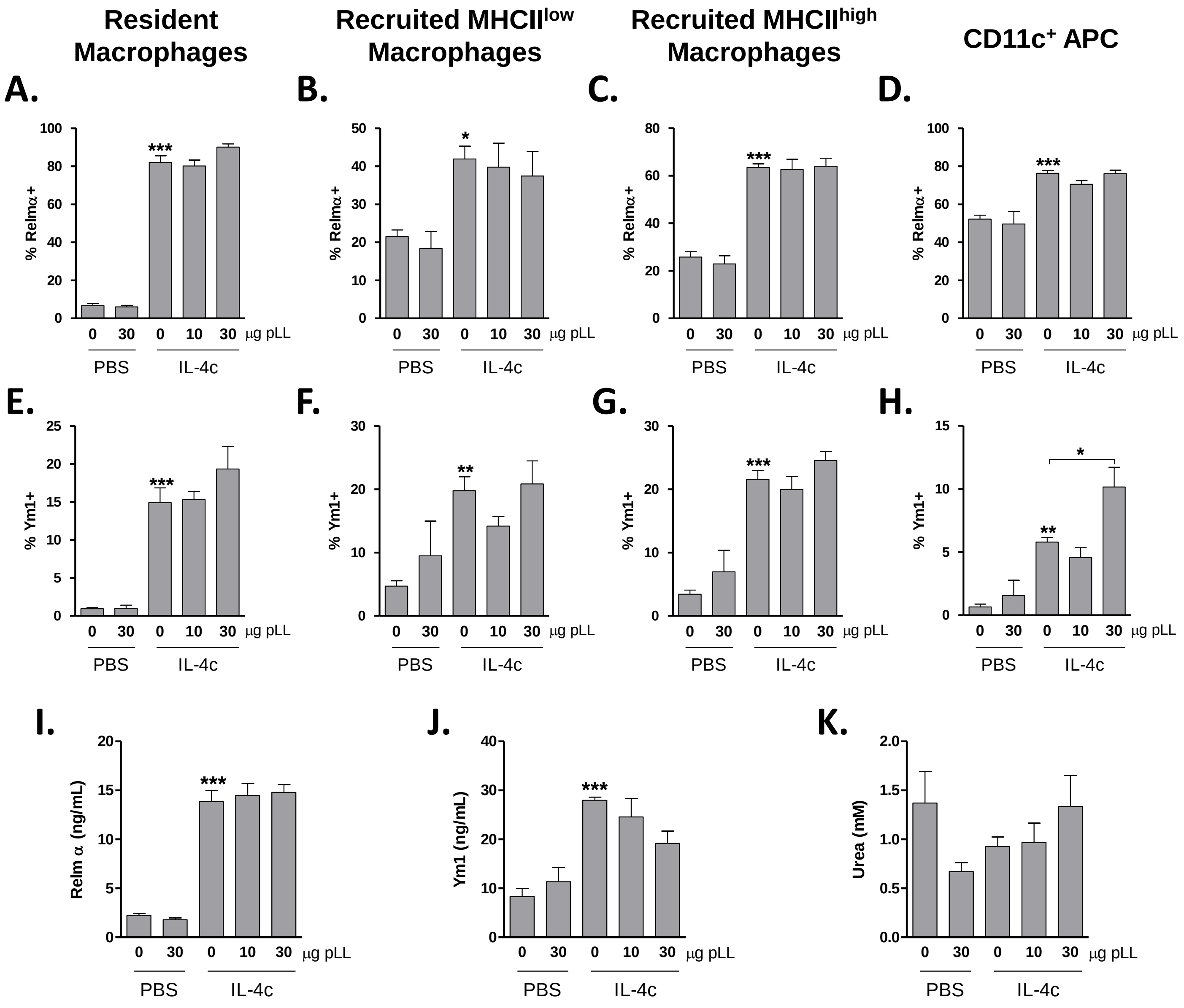
Resident Macrophages
Recruited MHCII^{low} Macrophages
Recruited MHCII^{high} Macrophages
CD11c^{+} APC
A.
B.
C.
D.
% Relmα+
***
*
***
***
0 30 0 10 30 μg pLL
PBS
IL-4c
E.
F.
G.
H.
% Ym1+
***
**
***
**
*
0 30 0 10 30 μg pLL
PBS
IL-4c
I.
Relm α (ng/mL)
***
J.
Ym1 (ng/mL)
***
K.
Urea (mM)
0 30 0 10 30 μg pLL
PBS
IL-4c

Figure S6. Peritoneal injection of pLL does not affect IL-4c-driven alternative activation of pleural cells. Mice were treated as described in Figure S1. Twenty-four hours post injection the proportion of pleural tissue resident macrophages, recruited $MHCII^{low}$ and $MHCII^{high}$ macrophages, and $CD11c^{+}$ APC expressing Relm α (**A-D**) or Ym1 (**E-H**) was assessed by flow cytometry. Also, the concentration of secreted Relm-α (**I**) and Ym1 (**J**), as well as Arginase activity in terms of urea formed in an *in vitro* reaction (**K**) were measured in the pleural lavage fluid. Bars depict mean and SEM of 5 animals per group and are representative of two independent experiments (except that the increase in the % of $Ym1^{+}$ cells in the $CD11c^{+}$ APC gate caused by 30 µg pLL in mice also administered IL-4c was not reproducible). * p≤0.05; ** p≤0.01; *** p≤0.001 (asterisks not associated with connecting lines represent differences with respect to mice injected with PBS only).

Figure S7

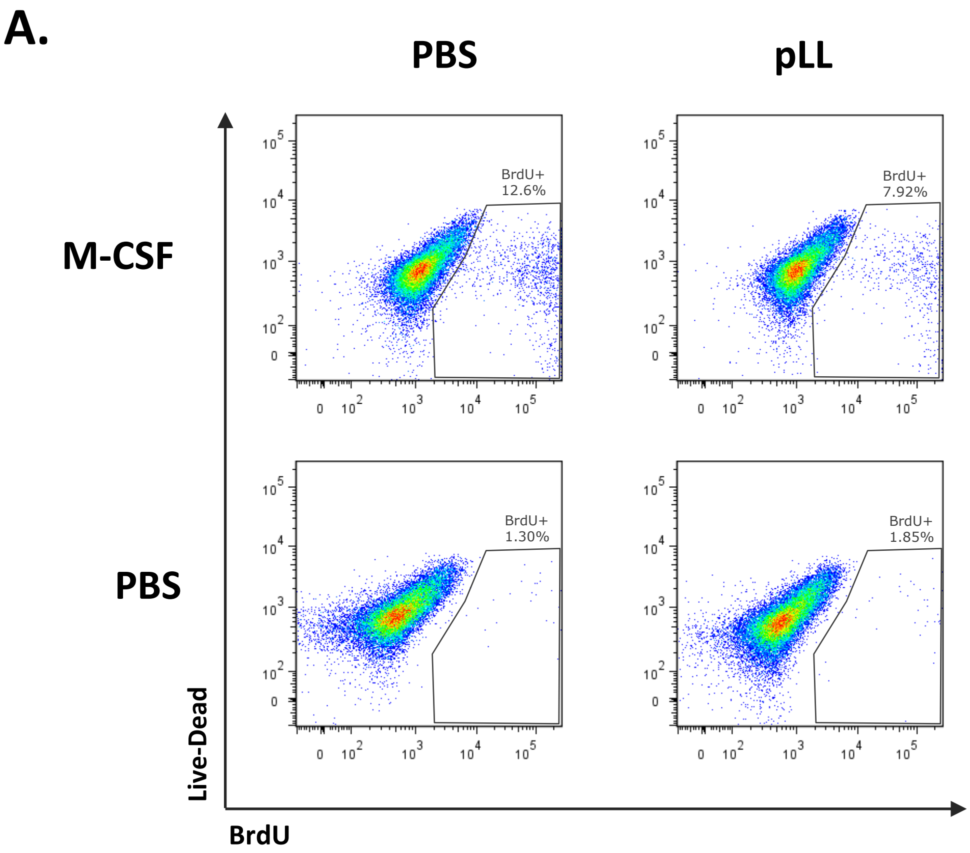
A.
PBS
pLL
M-CSF
PBS
BrdU+
12.6%
BrdU+
7.92%
BrdU+
1.30%
BrdU+
1.85%
Live-Dead
BrdU

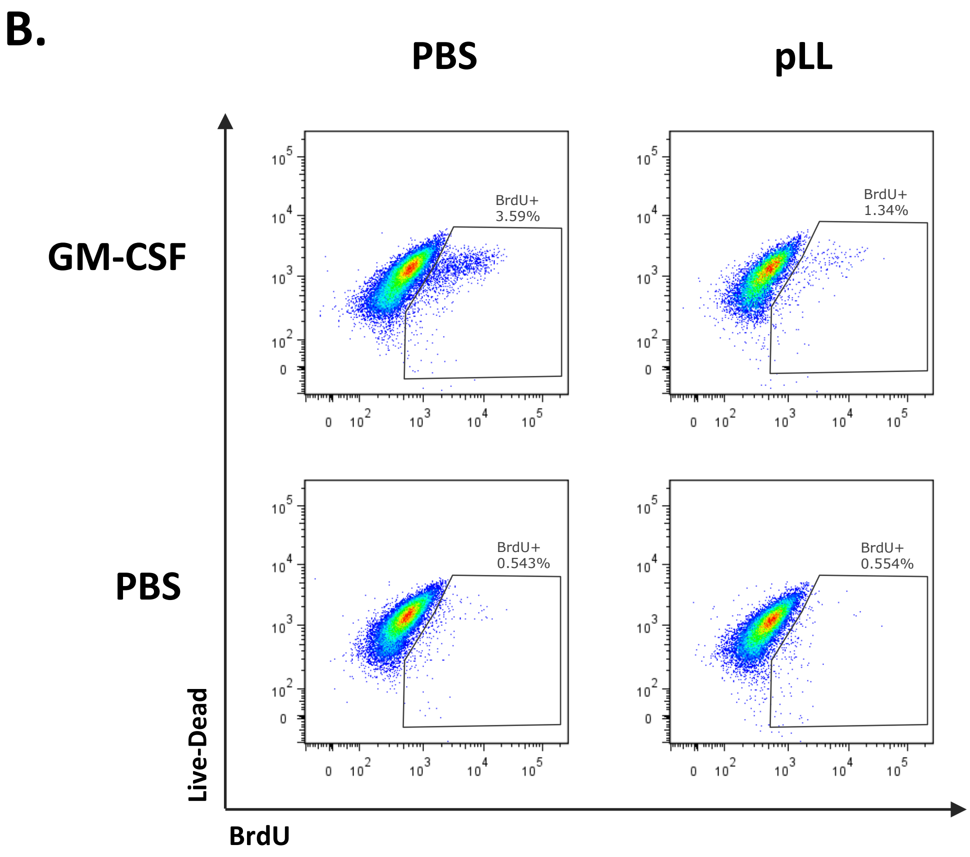
B.
PBS
pLL
GM-CSF
PBS
BrdU+
3.59%
BrdU+
1.34%
BrdU+
0.543%
BrdU+
0.554%
Live-Dead
BrdU

Figure S7. Representative dot-plots showing BrdU incorporation in macrophages incubated with M-CSF and in BMDCs incubated with GM-CSF, in the absence or presence of pLL. The results correspond to representative wells of the experiments shown in Figure 4, A and B.

Figure S8

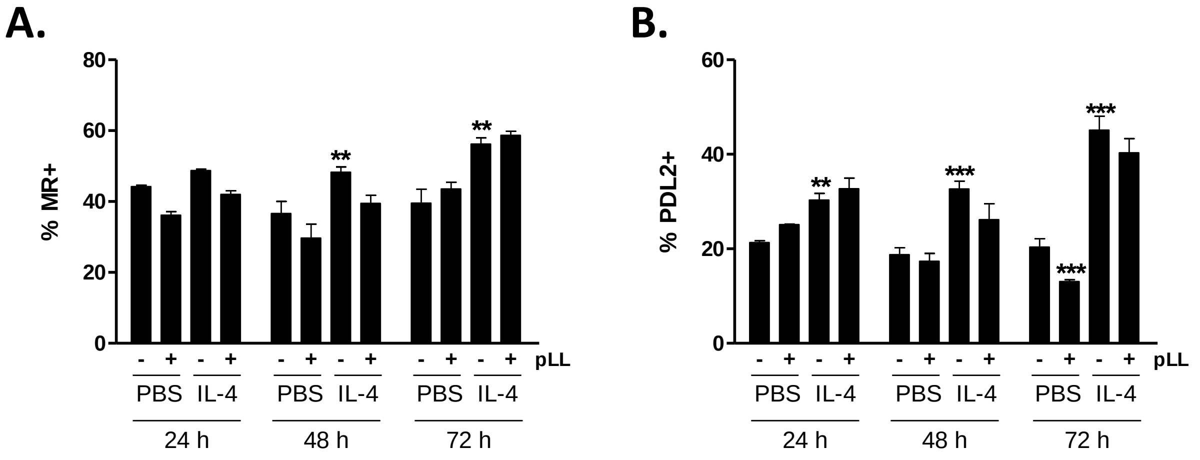
A.
80
60
40
20
0
% MR+
**
**
- + - + - + - + - + - + pLL
PBS IL-4 PBS IL-4 PBS IL-4
24 h 48 h 72 h
B.
60
40
20
0
% PDL2+
**
***
***
***
- + - + - + - + - + - + pLL
PBS IL-4 PBS IL-4 PBS IL-4
24 h 48 h 72 h

Figure S8. Exposure to pLL *in vitro* has no effect on the expression of mannose receptor or PDL2 induced by IL-4 in BMDCs. BMDCs were stimulated with IL-4 and in the absence or presence of pLL (25 μg per million cells) for the indicated times, and then analysed by flow cytometry for expression of the alternative activation markers mannose receptor (MR, CD206) (**A**) and PDL2 (**B**). Statistically significantly differences (by two-way ANOVA) are indicated: * $p \leq 0.05$; ** $p \leq 0.01$; *** $p \leq 0.001$ (asterisks not associated with connecting lines represent differences with respect to cells treated with medium only). The data are representative of two independent experiments.

Figure S9

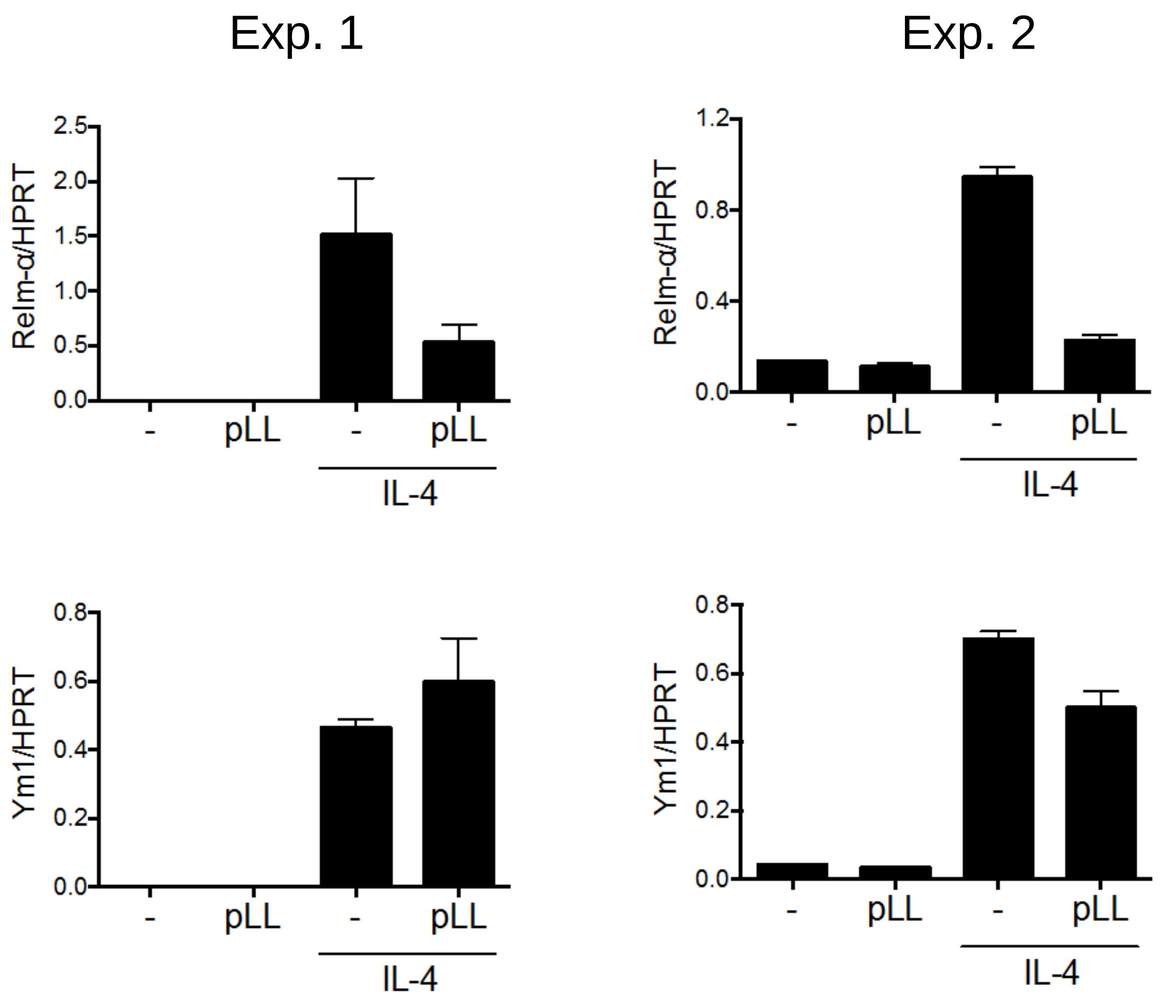
Exp. 1
Exp. 2
Relm-α/HPRT
Ym1/HPRT
2.5
2.0
1.5
1.0
0.5
0.0
1.2
0.8
0.4
0.6
0.2
-
pLL
IL-4

Figure S9. Exposure to pLL inhibits IL-4-driven up-regulation of Relm-α but not Ym1 at the transcriptional level in BMDMs. BMDMs were incubated with medium alone, IL-4 (20 ng/mL), pLL (150 µg dry mass per million cells) or pLL and IL-4. Six hours later, mRNA levels of mRNA coding for Relm-α and Ym1 were measured by reverse transcription/quantitative PCR, with normalisation over HPRT. Mean plus SEM of duplicate wells are plotted. The results of two independent experiments are presented.

Figure S10

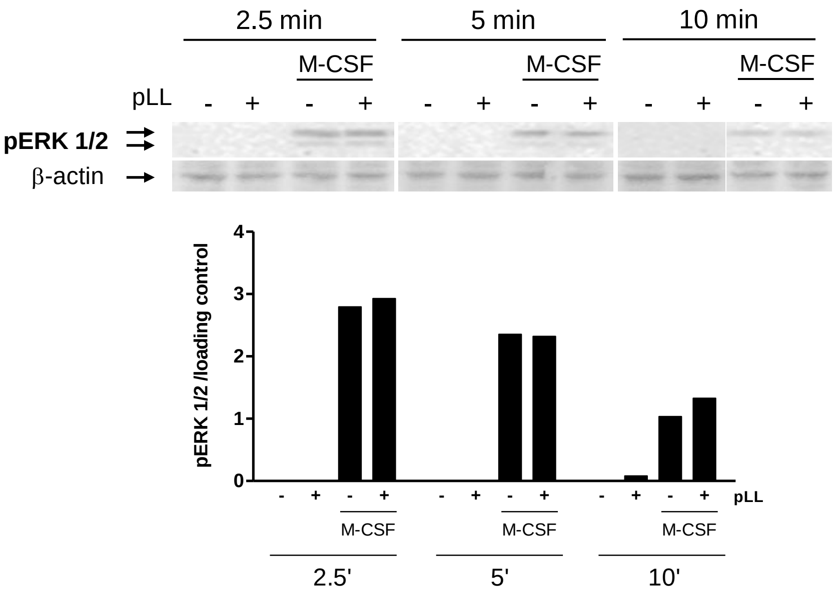

2.5 min
5 min
10 min
M-CSF
M-CSF
M-CSF
pLL - + - + - + - + - + - +
pERK 1/2
β-actin
pERK 1/2 /loading control
0
1
2
3
4
- + - + - + - + - + - + pLL
M-CSF
M-CSF
M-CSF
2.5'
5'
10'

Figure S10. Exposure to pLL *in vitro* does not alter ERK phosphorylation in macrophages. ThioMΦ were stimulated with M-CSF in the absence or presence of pLL and cells were lysed at the indicated times for analysis of p-ERK levels. Levels of pERK were normalised with respect to the Ponceau-stained β-actin band in each lane, and plotted besides the Western blot results. An experiment representative of two independent experiments is shown. Full-length blots are shown in Figure S12.

Figure S11

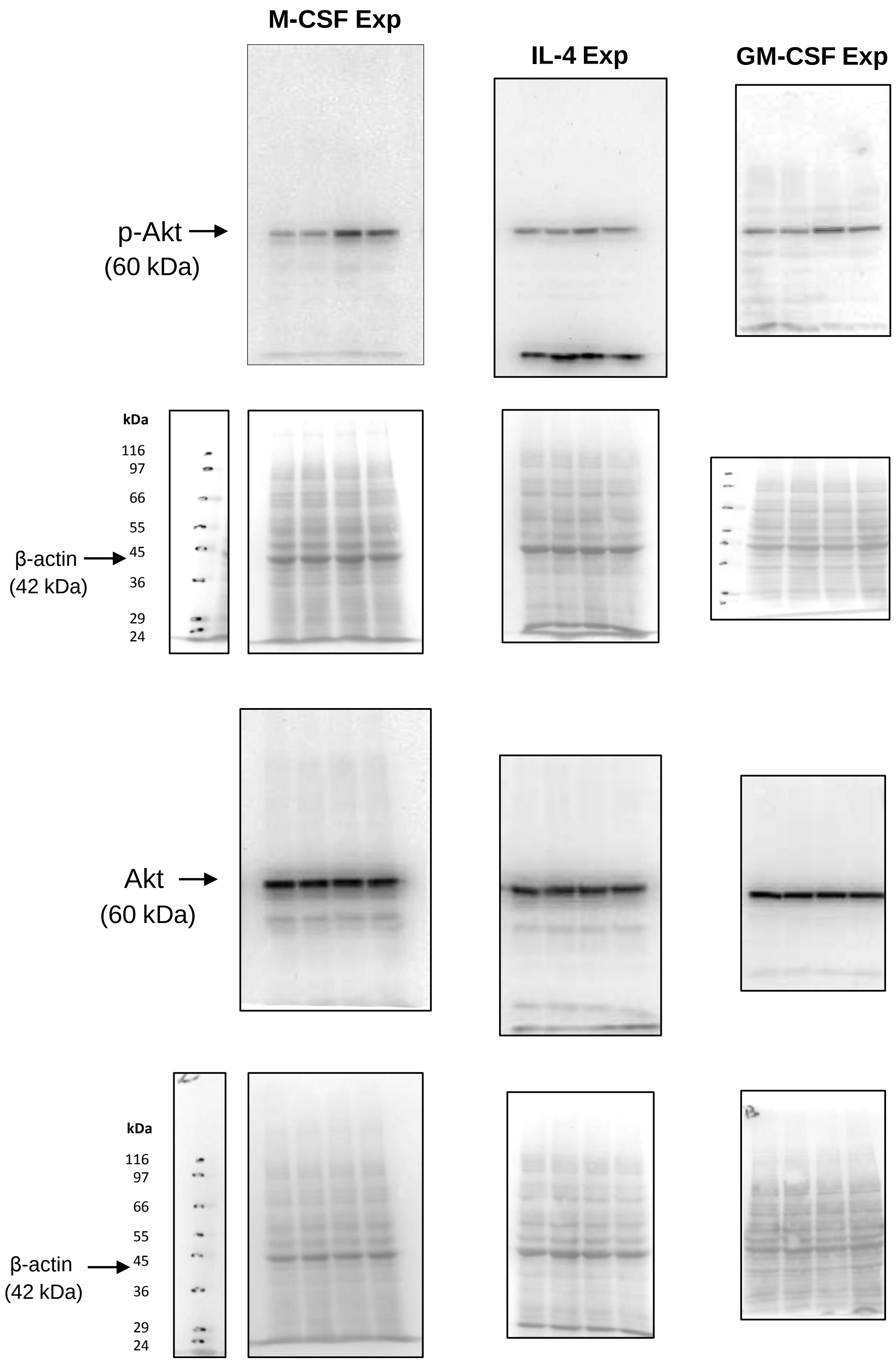
M-CSF Exp
IL-4 Exp
GM-CSF Exp
p-Akt
(60 kDa)
kDa
116
97
66
55
45
36
29
24
β-actin
(42 kDa)
Akt
(60 kDa)
kDa
116
97
66
55
45
36
29
24
β-actin
(42 kDa)

Figure S11. Uncropped Western blots corresponding to the results shown in Figure 6.

Figure S12

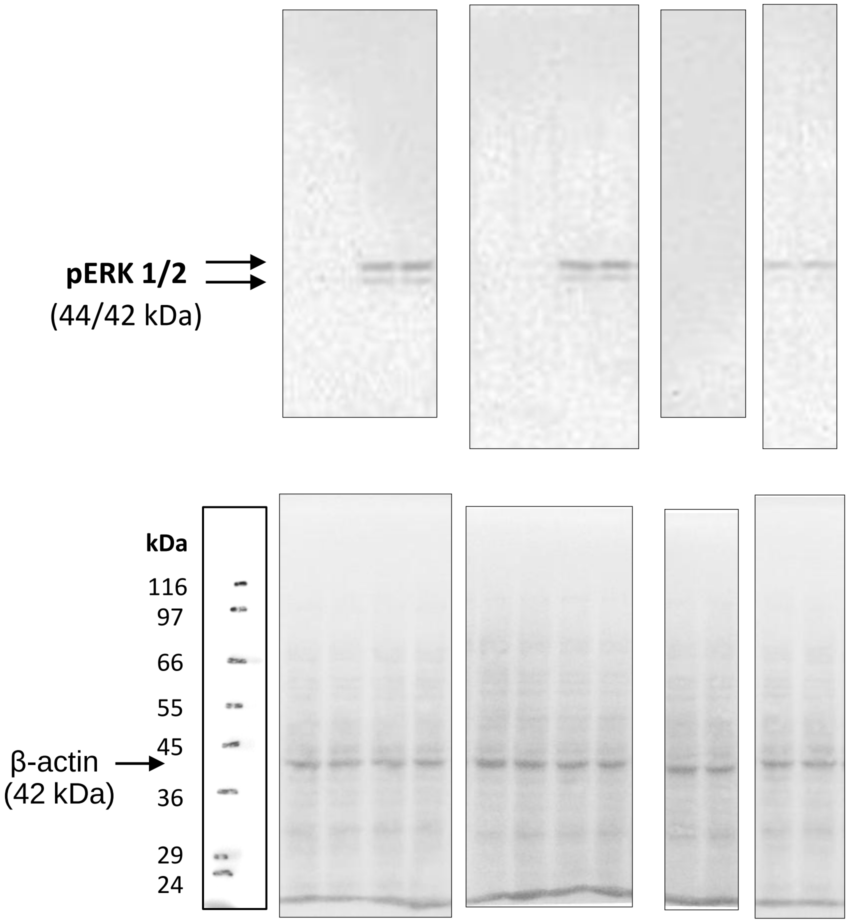

Figure S12. Uncropped Western blots corresponding to the results shown in Figure S10.
